# The influence of experience and modality of presentation (online vs. offline) on hypnotizability

**DOI:** 10.3389/fpsyg.2023.1293070

**Published:** 2024-02-28

**Authors:** Björn Rasch, Maren Jasmin Cordi

**Affiliations:** ^1^Division of Cognitive Psychology, Department of Psychology, University of Fribourg, Fribourg, Switzerland; ^2^Sleep and Health Zurich, University of Zurich, Zürich, Switzerland

**Keywords:** hypnotizability, experience, modality, online vs. offline, hypnosis

## Abstract

**Introduction:**

Hypnotizability is conceptualized as a stable personality trait describing the ability to respond to suggestions given under hypnosis. Hypnotizability is a key factor in explaining variance in the effects of hypnotic suggestions on behavior and neural correlates, revealing robust changes mostly in high hypnotizable participants. However, repeated experience and training have been discussed as possible ways to increase willingness, motivation, and ability to follow hypnotic suggestions, although their direct influence on hypnotizability are still unclear. Additionally, it is important whether hypnotizability can be assessed reliably online.

**Methods:**

We investigated the influence of the degree of experience with hypnosis and the presentation mode (online versus live) on the stability of hypnotizability in two groups of 77 and 102 young, healthy students, respectively. The first group was tested twice with the Harvard Group Scale of Hypnotic Susceptibility (HGSHS) after two weeks. During this period, participants either repeatedly listened to a hypnosis or trained on a progressive muscle relaxation or served as waitlist control group. In the secondgroup, participants performed both an online or offline version of the HGSHS, with varying time intervals (1–6 weeks).

**Results:**

Contrary to our expectations, hypnotizability declined from the first to second assessment in the first group. The reductionwas most prominent in initially highly hypnotizable subjects and independent of the experience intervention. We observed a similar reduction of hypnotizability in the second group, independent of presentation modality. The reduction was again driven by initially highly hypnotizable subjects, while the scores of low hypnotizable subjects remained stable. The presentation modality (online vs. offline) did not influence HGSHS scores, but the test–retest reliability was low to moderate (r_tt_ = 0.44).

**Discussion:**

Our results favor the conclusion that generally, hypnotizability is a relatively stable personality trait which shows no major influence of preexperience or modality of assessment. However, particularly highly hypnotizable subjects are likely to experience a decline in hypnotizability in a retest. The role of the concrete assessment tool, psychological factors, and interval length are discussed. Future studies should replicate the experiments in a clinical sample which might have higher intrinsic motivation of increasing responsiveness toward hypnotic interventions or be more sensitive to presentation mode.

## 1 Introduction

There is quite a long history about finding a well-accepted definition of hypnosis (see Green et al., 2005). One suggestion is to define hypnosis as “a state of consciousness involving focused attention and reduced peripheral awareness characterized by an enhanced capacity for response to suggestion” (Elkins et al., 2015, p. 6), even though this definition is not without criticism, as will be discussed later (see Lynn et al., 2015). Increasing scientific evidence exists for the efficacy of applying hypnotic interventions as treatment for a wide range of disorders, illnesses, or other health purposes. It has been shown to reduce pain (Thompson et al., 2019) and post-menopausal hot flashes (Elkins et al., 2013), lower depressive symptoms (Milling et al., 2018) or anxiety (Valentine et al., 2019), and deepen sleep (Cordi et al., 2014, 2015, 2020; Besedovsky et al., 2022). While most meta-analyses report high overall effect sizes between 0.67 to 0.79 (Montgomery et al., 2000; Milling et al., 2018; Valentine et al., 2019), treatment success can depend strongly on the degree of hypnotizability. Hypnotizability can be defined as the general tendency to respond to hypnosis and hypnotic suggestions (Gur, 1978) or, including the subjective experience, describe “an individual’s ability to experience suggested alterations in physiology, sensations, emotions, thoughts, or behavior during hypnosis” (Elkins et al., 2015, p. 6). Subjects with a high hypnotizability benefit from hypnotic treatments with a large effect size of 1.16; effect sizes of medium hypnotizability are around 0.64 (Montgomery et al., 2000). In contrast, low hypnotizable “non-responders” show negligible (Montgomery et al., 2000; Cordi et al., 2015) or even negative reactions to hypnotic interventions (Cordi et al., 2014). Large correlations between the amount of hypnotic treatment benefits and hypnotizability of *r* = 0.50 support this observation (Liossi et al., 2006). Such results suggest that highly hypnotizable subjects have a greater chance to benefit from hypnosis than low hypnotizable subjects. Some researchers thus highlight hypnotizability as a main predictor for hypnotic responsiveness and treatment success (e.g., Barabasz and Perez, 2007). Contrary, other authors state in their reviews on hypnotizability and treatment outcome that the association between hypnotizability and treatment outcome is only mixed (Lynn et al., 2003; Wofford et al., 2023). According to this view, the only exception was pain treatment for which associations between degree of hypnotizability and treatment success have been quite consistent.

As hypnotizability appears to play an important role at least in some treatment areas, it is an important question whether hypnotizability is a stable individual trait or whether it can be increased by repeated exposure and training. Most researchers define or compare hypnotizability with other personal trait variables (Milling et al., 2006; Barabasz and Perez, 2007) and assume a significant contribution of genetic factors (Morgan, 1973; Moss and Willmarth, 2019). These assumptions are strengthened by longitudinal studies which reported long-term stability across test intervals of 8 to 12 years (Morgan et al., 1974) or 25 years (Piccione et al., 1989). In spite of this evidence for the long-term stability of hypnotizability, attempts to enhance hypnotizability by providing information, strategies to follow the suggestions, and observational learning have proven successful for subjective and behavioral measures (Gorassini and Spanos, 1986). This “Carleton Skills Training Package” was retested later by Bertrand et al. (1993) who confirmed the increments in hypnotizability across different scales measured in two posttests, 2 and 3 weeks after training. Other authors confirmed improvements after hypnotizability training in objective but not subjective scores of hypnotizability, as measured by observations of overt reactions (Bates and Brigham, 1990). In sum, explicit training of hypnotizability appears to be possible. For individuals of low hypnotizability, it might even merely require more experience (e.g., more hypnotic sessions) to improve their ability to respond to hypnotic suggestions (Elkins, 2021). For example, Kaczmarska et al. (2020) reported improvements in hypnotizability after a minimum of three sessions of hypnotherapy. However, others reported significant decrements in hypnotizability scores after repeated confrontation with hypnotic inductions (Barber and Calverley, 1966; Fassler et al., 2008). Taken together, despite reports of long-term stability of hypnotizability, evidence suggests that hypnotic responsiveness can be modified, probably even by mere exposure to hypnosis. The first aim of our study was thus to test to what extent experience with hypnosis boosts the ability to respond to hypnotic induction, as reflected in measures of hypnotizability.

A second aim of our study was to examine the influence of presentation mode on hypnotizability scores. As group sessions in presence are resource-demanding, pre-screening hypnotizability using online assessments could be a time- and cost-effective alternative if measures are reliable. Previous research showed that delivering hypnosis by audiotape or an experimenter did not systematically influence hypnotizability scores in experiments (Fassler et al., 2008). A recent study by Palfi et al. (2020) directly examined the comparability of online vs. offline assessments of hypnotizability. A sample of 71 young and healthy students were assessed twice using the audio version of the Sussex Waterloo Scale of Hypnotizability (SWASH; Lush et al., 2018). All participants were tested in groups offline first. Afterwards, 26 participants were again assessed offline in a standardized room with the experimenter present, but in individual sessions. The other 45 participants were assessed in individual sessions alone in their rooms at home (online). The study revealed comparable levels of responsiveness in both the offline and online version. The authors concluded that online procedures of hypnotizability assessments are a consistent and reliable alternative (Palfi et al., 2020). However, to our knowledge, despite recent increases in usage of online surveys and interventions since COVID-19, this is the only study that directly tested the impact of presentation mode on hypnotizability. In addition, the order of offline vs. online assessment was not randomized in this study. Thus, replication of these findings and the generalization to the widely used Harvard Group Scale of Hypnotic Susceptibility (HGSHS) is important and necessary.

77 participants and created different degrees of hypnotic experience in the two-week interval between two assessments of hypnotizability by the HGSHS (Shor and Orne, 1963). During the two weeks, we asked subjects to either listen to hypnotic suggestions or perform progressive muscle relaxation (PMR) on a daily basis, in a between-subjects design. The latter controls for the influence of a similarly relaxing but not explicitly hypnotic technique. Finally, we compared both groups to a waitlist control condition without intervention. We expected that increased amounts of experience with hypnosis would enhance hypnotizability as measured by the HGSHS. To test our second aim, a separate group of 102 healthy young participants was assessed twice with the HGSHS, with an interval of one to six weeks. In a counter-balanced order according to a within-subjects design, they were confronted with an online and an offline version in group sessions. We predicted that the presentation modality does not have a major influence on the assessment of hypnotizability. However, we hypothesized that the second assessment should reveal generally higher hypnotizability scores due to the increased experiences of the participants, independent of presentation modality.

## 2 Materials and methods

### 2.1 Participants

A group of *n* = 77 students took part in the experience manipulation experiment (experiment 1, 60 females; age range 18–43; average age = 21.61, SD = 3.91). In the online/offline experiment (experiment 2), data of *n* = 102 subjects were analyzed (76 females, age range 18–55, average = 23.31, SD = 6.91). Recruitment in both studies was done with flyers, announcements, and calls in lectures of psychology at the University of Fribourg. During the first session, each subject provided informed consent. In both studies, inclusion criteria encompassed age 18 or above and good knowledge of German. Participants were compensated by 4.5 subject hours in the experience experiment and by 3 h in the online/offline experiment. In case of drop out, they were compensated proportionally. The ethical review board of the Department of Psychology, University of Fribourg, approved the study (approval No 54).

#### 2.1.1 Randomization

In experiment 1, subjects were randomly assigned to one of three conditions: *n* = 26 subjects were asked to perform progressive muscle relaxation daily, *n* = 26 to listen to the hypnotic suggestions, and *n* = 25 were assigned to a waitlist control group without intervention during the 2-week period. Those groups neither differed in age (*p* = 0.55) nor suggestibility at time point 1 (Score: *p* = 0.86 / depth: *p* = 0.88). Sex was equally distributed across the conditions (*p* = 0.58).

In experiment 2, subjects were randomly assigned to two between-subjects order conditions. *n* = 51 were first tested online, then offline (online first group). The second group of *n* = 51 participants were tested offline first and online in the second session (offline first group). Sex was equally distributed across the conditions (*p* = 0.17). The group tested offline first was on average older (24.78 ± 8.99) than the other group (21.84 ± 3.35), unpaired *t*-test *t*(63.62) = 2.19, *p* = 0.03, d = 0.43. This difference was mainly due to one person aged 55 in the offline first group. Excluding this person would result in equal age groups (*p* = 0.06). We refrained from this option for our analyses, as the Pearson correlation between age and HGSHS scores in session 1 was close to zero [*r*(100) = 0.06, *p* = 0.58], also when both samples were merged [*r*(174) = 0.04, *p* = 0.61].

### 2.2 Procedure

**Figure 1:**
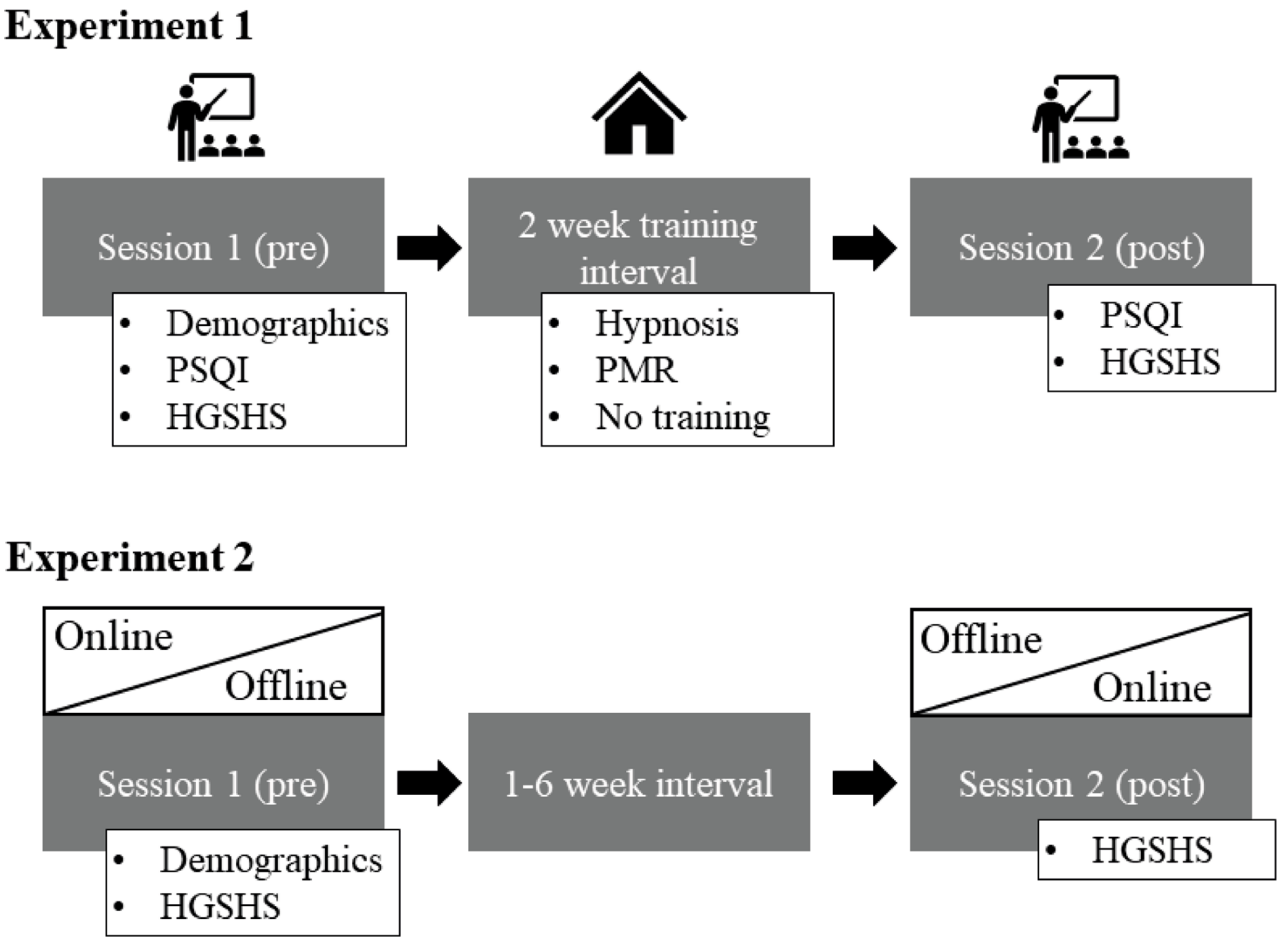
The session flow of experiment 1 in the upper row and experiment 2 in the lower part of the picture. In experiment 1, session 1 and 2 took place at the university while the 2-week intervention training took place in the subject’s homes. In experiment 2, the order of online versus offline presentation of the hypnotizability measure was randomized. It either took place at the university first (offline) and online second (at the subjects’ homes) or reversed. PSQI, Pittsburgh Sleep Quality Index; HGSHS, Harvard Group Scale of Hypnotic Susceptibility; PMR, Progressive Muscle Relaxation.

Data collection in experiment 1 consisted of two in-house sessions (pre and post) at the University of Fribourg and were separated by the interval of 2 weeks in which the intervention took place (see upper part of Figure 1). Each session took around 90 min and was conducted in groups of different sizes. In the first session, subjects filled in the demographic questionnaire, HGSHS, and Pittsburgh Sleep Quality Index (PSQI, Buysse et al., 1989), and received instructions for the intervention interval. After two weeks, the second session included another measure of the HGSHS and PSQI and ended by compensating the subjects. The two intervention weeks took place at the subjects’ homes. For those, subjects were instructed to listen to the assigned audio file (hypnosis or PMR) on a daily basis. They received a download link to install the according audio file on their mobile device. The waitlist control group did not receive any instructions.

Data in experiment 2 were collected in two sessions, out of which one took place online and the other offline, in a randomized order, separated by 1–6 weeks (see lower part of Figure 1). In each session, subjects answered the HGSHS and a demographic questionnaire. Other questionnaires assessed in the sessions are not relevant for this work [Mehrdimensionaler Befindlichkeitsbogen (MDBF), Positive and Negative Affect Schedule (PANAS), and Creative Achievement Questionnaire (CAQ)]. The offline sessions took place in groups of different sizes in a quiet room at the University of Fribourg with the experimenter present. The online sessions took place via video call on Microsoft Teams. Each participant was asked to sit in a quiet room, switch off the microphone but switch on the camera for security reasons. In both conditions, the German version of the HSGSHS audio recording was played via loudspeakers before subjects self-rated the items in the according booklet. After the second session, subjects were compensated.

### 2.3 Questionnaires

#### 2.3.1 Hypnotizability

Subjective hypnotizability was assessed with the Harvard Group Scale of Hypnotic Susceptibility, Form A (Shor and Orne, 1963) in its German translation (Bongartz, 1985). This is a standardized self-assessment form which is frequently used in hypnosis research and can be used in groups of unlimited sizes (Angelini et al., 1999). Its test–retest reliability had previously been tested in a Polish sample (sessions on the same day *r* = 0.69, 8 weeks apart *r* = 0.58) (Siuta, 2010). It includes a standardized audio file with a hypnotic induction, followed by several hypnotic suggestions. The latter are in increasing difficulty from simple motor/kinesthetic responses to acoustic hallucinations and cognitive items (amnesia and a posthypnotic suggestion). The score is calculated by counting each item that the subject followed in the questionnaire. In a second part of the booklet, subjects indicate how deep they felt in a hypnotic state during each of the 12 suggestions on a Likert scale of 1 to 10. The mean of these items was taken as trance depth. Taking both samples together (excluding *n* = 3 for which 1 item of the HGSHS was missing data), those two measures correlate significantly [*r*(174) = 0.58, *p* < 0.001] at measurement time 1 and *r*(174) = 0.67, *p* < 0.001 at time 2. The suggested cut-off values refer to the first part of the questionnaire (i.e., the objective scores). Six points or less indicate low to medium hypnotizability, while 7 or more categorize medium to high hypnotizability. Together, *n* = 29 male participants and *n* = 61 female participants were considered medium-to-low hypnotizable in session 1 and *n* = 14 male and *n* = 72 female participants medium-to-high. For reasons of simplicity, we will refer to the groups as low and high hypnotizable in the following. Male participants generally scored lower on the HGSHS scores (5.84 ± 0.29) than female participants (6.62 ± 0.19), unpaired *t*-test *t*(174) = −2.11, *p* = 0.036, d = 3.58 but not in hypnotic depth (4.76 ± 0.21 and 5.07 ± 0.13, for male and female participants, respectively, *p* = 0.24). Chi^2^ tests indicated a sex bias in HGSHS scores [Chi^2^(1) = 6.06, *p* = 0.014]. Even though Cramer’s V is significant (Cramers *V* = 0.19, *p* < 0.001), it is below 0.3 and hence, the association is rather small and mainly due to a lower than expected number of male participants in the high hypnotizable group. An additional item at the beginning of the questionnaire asks for previous experience with hypnosis and experience with relaxation and was coded with 1 for experience and 0 for no experience.

#### 2.3.2 Sleep protocol

In order to detect possible influences of the hypnotic suggestions or the progressive muscle relaxation intervention on subjective sleep, we assessed subjective sleep quality with a daily sleep protocol. This data will be published elsewhere. It however included an item asking whether the training (i.e., PMR or hypnosis) was accomplished. To assess the degree of commitment to the instructions, we summed up how often subjects indicated usage of the file during the 14 days. Commitment is used as a covariate in the ANCOVA.

#### 2.3.3 Sleep quality

To assess the influence of hypnotic suggestions or PMR on sleep, we measured the Pittsburgh Sleep Quality Index (PSQI, Buysse et al., 1989) before and after the training interval in all subjects. We calculated the difference in subjective sleep quality scores from post to pre-training to assess the improvement across training. We included this variable as a covariate in the ANCOVA to control for a potential influence of training success on the changes in hypnotizability.

#### 2.3.4 Demographics

The demographic questionnaires assessed Sex, age, size, weight, and existence of diagnosed neurologic or psychiatric issues, medication, or substance use.

#### 2.3.5 Experience manipulation

The hypnosis group was given access to the audio file containing the hypnotic suggestions for increasing sleep depth that we had previously used in other studies (e.g., Cordi et al., 2014, 2015, 2020). Participants were asked to listen to the file during falling asleep each evening during the 2-week intervention period. They were allowed to fall asleep at any time during or after the hypnosis. The file includes a 14-min recording of a male, gentle voice, speaking slowly and softly. It contains 4 min of hypnotic induction, followed by suggestions to sleep deeper and relax.

The PMR (progressive muscle relaxation) group received access to an audio file including a 20-min guided PMR session. We had used a long version of this file previously (Combertaldi et al., 2021). A male speaker guides through the exercise with a soft voice, while relaxing music is played in the background.

### 2.4 Statistical analysis

We calculated 3 × 2 × 2 repeated measure ANOVAs with experience (hypnosis, PMR, and control) and hypnotizability (high vs. low) as the between-subjects factors and measurement time (pre vs. post) as the within-subjects factor. To estimate the influence of covariates, we re-analyzed the upper ANOVA including the additional between-subjects factor experience with hypnosis or experience with relaxation. Additionally, we analyzed an ANCOVA including training success, measured as the difference between PSQI after training – PSQI before training as covariate. To assess the influence of previous experience with hypnosis or relaxation, we analyzed a 3 × 2 × 2 × 2 ANOVA including this additional between-subjects factor. Moreover, we calculated Pearson linear correlations and Cronbach’s alpha between the two scales of the HGSHS.

To test the on/offline effect, we calculated a paired *t*-test between online vs. offline measured hypnotizability scores and depth of hypnosis, indicating effect sizes using Cohen’s d. Moreover, we calculated a Pearson correlation between the scores measures with the two modalities to measure the degree of their correspondence. To test the influence of measurement time, we resorted the HGSHS scores to assessment at session 1 vs. session 2, independent from modality or order of presentation. Moreover, we included the between-subjects factor hypnotizability as measured in the first session (high vs. low) into a 2 × 2 repeated measures ANOVA with the between-subjects factor measurement time (session 1 vs. 2). The results of the complete models can be found in the Supplementary material.

Generally, we followed up on significant results with *post hoc t*-tests for independent samples or paired *t*-tests. For all analyses, we tested whether the statistical assumptions were met. If Levene’s test indicated unequal variances, we used the corrected *t*-value and degrees of freedom. In case of non-significant post-hoc *t*-tests, we calculated the Bayes-Factor BF_0/1_ for the comparison between H0 (no difference) versus H1 (difference). Values greater than 3 are taken as evidence in favor of the nominator, i.e., the H0 hypothesis (van Doorn et al., 2021). Alpha power was set to *p* = 0.05. Averages are reported as mean ± standard error of the mean (SEM), if not indicated otherwise.

## 3 Results

### 3.1 Experiment 1: impact of experience on hypnotizability

**Figure 2:**
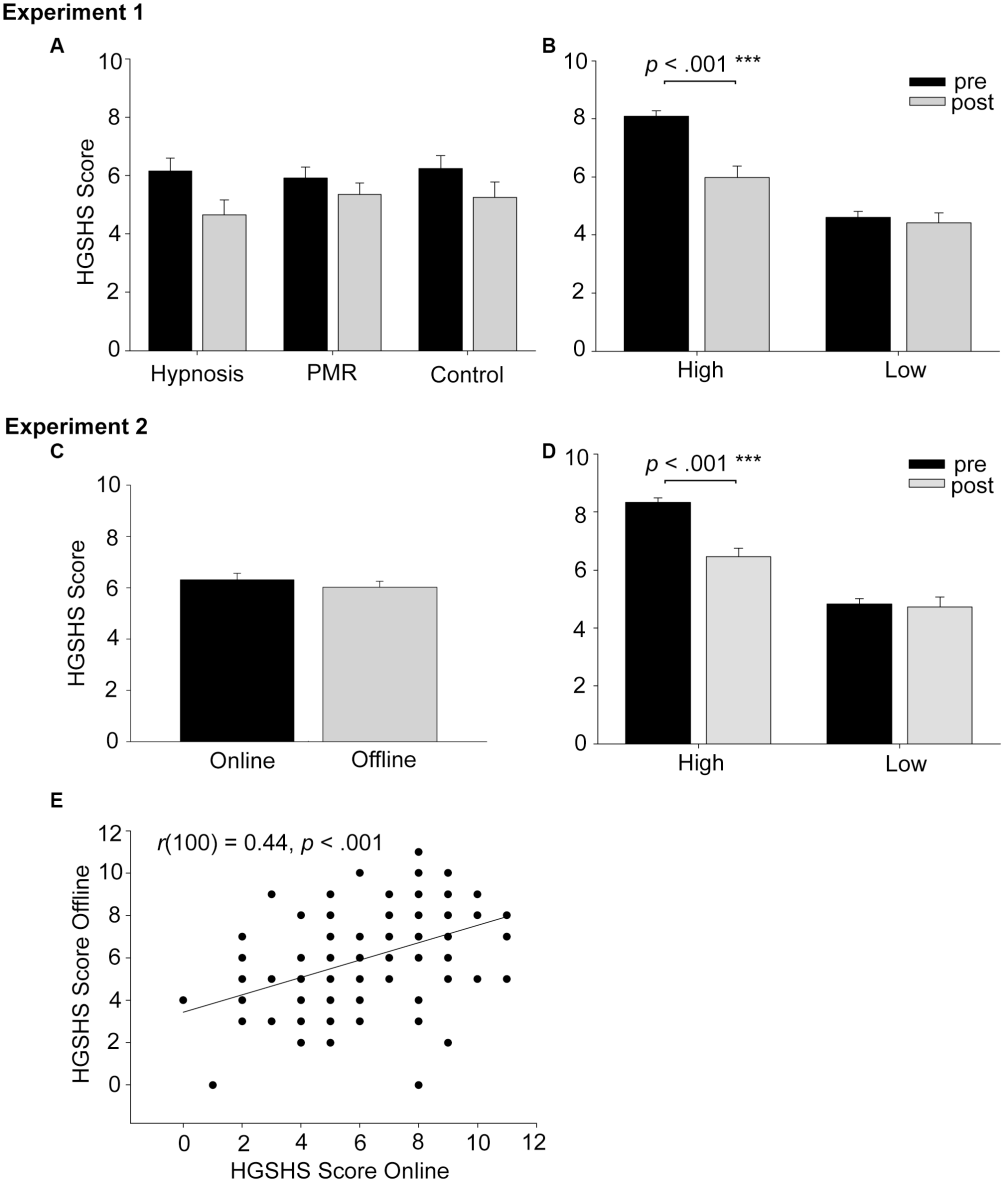
Shows the results of experiment 1 (upper row) and experiment 2 (lower part). Panel **(A)** shows that independent from intervention, scores generally decreased from pre- (session 1) to post-intervention (session 2). Panel **(B)** shows that hypnotizability measures significantly diminished from pre to post measure only in high but not low hypnotizable subjects. This was independent from success of the hypnotic intervention to improve subjective sleep quality. Panel **(C)** displays the means of HGSHS scores measured online vs. offline. The modality of measuring hypnotizability does not influence the outcome. Panel **(D)** displays the results including hypnotizability as assessed in the first measure as a factor, which resulted in the same results pattern as in experiment 1: the scores of high hypnotizable subjects significantly diminished from the first to second session, while low hypnotizable participants remained stable. Panel **(E)** shows that online and offline measure scores highly correlated [*r*(100) = 0.44, *p* < 0.001], which is however a low to moderate test–retest reliability.

After the two-week intervention period, HGSHS scores differed significantly, as indicated by a significant main effect of measurement time (3 × 2 × 2 repeated measure ANOVA, *F*(1, 71) = 24.09, *p* < 0.001, eta^2^ = 0.25). Contrary to our prediction, the scores of hypnotizability significantly decreased from 6.10 ± 2.11 (average score) in the first assessment of the HGSHS to 5.08 ± 2.42 in the second assessment after two weeks. Separation of participants in high (HGSHS ≥7) and low hypnotizable individuals (HGSHS <7) based on the first assessment revealed that the reduction was most prominent in high hypnotizable subjects, who significantly decreased from 8.09 ± 0.19 to 5.97 ± 0.40, paired *t*-test *t*(32) = 5.60, *p* < 0.001, *d* = 1.13. Low hypnotizable participants did not significantly change on their scores across the interval [*t*(43) = 0.75, *p* = 0.46, *d* = 0.1; means: pre: 4.61 ± 020, post: 4.41 ± 0.35]. There was substantial evidence in favor of an absence of an effect (BF0/1 = 6.46). This difference was reflected in a significant interaction between measurement time and hypnotizability [*F*(1, 71) = 15.45, *p* < 0.001, eta^2^ = 0.18], see Figure 2B. Contrarily, and against our expectation, the interaction between intervention and measurement time was however non-significant [*F*(2, 71) = 0.55, *p* = 0.58, eta^2^ = 0.02], indicating that the degree of experience did not influence the change in hypnotizability scores (see Figure 2A). All other main effects and interactions were non-significant (*p* > 0.30, see Supplementary material).

#### 3.1.1 Covariate analysis

Those results did not change when training success, quantified as difference in subjective sleep quality across the interval (PSQI post - pre) was considered as a covariate in the 3 × 2 × 2 ANCOVA [main effect for PSQI difference *F*(1, 63) = 0.42, *p* = 0.52, eta^2^ = 0.01]. Neither did the inclusion of the dichotomous factor experience with hypnosis or experience with relaxation as a factor in the 3 × 2 × 2 × 2 ANOVA change the results (main effect of experience with hypnosis, *F*(1, 63) = 0.99, *p* = 0.32, eta^2^ = 0.02, and main effect of experience with relaxation, [*F*(1, 63) = 1.22, *p* = 0.27, eta^2^ = 0.02]. Also, the correlation between PSQI difference and score difference was non-significant in the hypnosis group [*r*(23) = −0.18, *p* = 0.39] as well as in the PMR group [*r*(19) = 0.28, *p* = 0.22].

### 3.2 Experiment 2: online vs. offline modality of HGSHS assessment

We first tested the impact of the presentation modality on hypnotizability scores. Online measured hypnotizability was descriptively slightly higher (6.31 ± 0.24) compared with offline collected hypnotizability (6.02 ± 0.22), but this difference was not significant [paired *t*-test *t*(101) = 1.22, *p* = 0.23, *d* = 0.13; see Figure 2C]. There was substantial evidence in favor of an absence of effect (BF0/1 = 6.15). The same was true for depth of hypnosis [4.85 ± 0.16 and 4.87 ± 0.16, for online and offline, respectively, *t*(101) = −0.13, *p* = 0.90, *d* = 0.01]. There was strong evidence in favor of an absence of an effect (BF_0/1_ = 12.67). The observed statistical power to detect a medium-sized effect of *f* = 0.25 was over 99% with our sample of 102 participants. Thus, we were even able to exclude small-to-medium effect sizes from *f* = 0.15 with 80% certainty, safely excluding that the presentation modality induced small-to-medium differences in hypnotizability. However, we could not exclude the existence of small differences between online and offline versions of the HGSHS.

The test–retest correlation of the online vs. offline version of the HGSHS was highly significant, but only in a low to moderate range [*r*(100) = 0.44, *p* < 0.001, see Figure 2E]. Cronbach’s alpha for suggestibility measured online vs. offline was = 0.61. Similar correlations occurred for the assessments of depth of hypnotic trance [*r*(100) = 0.41, *p* < 0.001]. However, as test–retest reliability, this association should be considered too low for a reliable assessment, as acceptable reliability would begin at correlations of *r* > 0.7.

In a second step, we tested the influence of exposure on the assessments of hypnotizability, independent of presentation modality. Thus, we resorted hypnotizability scores into first and second measurement time, independent from modality. Confirming our results from experiment 1, hypnotizability scores were generally higher in the first measure (6.69 ± 0.21) than in the second measure (5.65 ± 0.23), (2 × 2 repeated measure ANOVA, *F*(1, 100) = 23.67, *p* < 0.001, eta^2^ = 0.19).

When separating participants again into high and low hypnotizable individuals, we observed a significant interaction with the factor low vs. high hypnotizability [*F*(1, 100) = 18.94, *p* < 0.001, eta^2^ = 0.16, see Figure 2D]. Similar to experiment 1, we again observed that the reduction in HGSHS scores was only significant for initially high hypnotizable subjects (8.33 ± 0.16 and 6.46 ± 0.29 for the first and second measure, respectively, paired *t*-test *t*(53) = 6.40, *p* < 0.001, *d* = 1.07). This comparison was non-significant in low hypnotizable subjects [4.83 ± 0.18 and 4.73 ± 0.33, *t*(47) = 0.38, *p* = 0.71, *d* = 0.05]. There was substantial evidence in favor of an absence of an effect (BF_0/1_ = 8.27).

#### 3.2.1 Explorative analysis

Testing whether the amnesia item in the HGSHS was particularly more difficult to meet in initially highly hypnotizable subjects, we ran an ANOVA with the within-subjects factor measurement time and the between-subjects factor hypnotizability on this item. It resulted in a main effect of time [*F*(1, 100) = 6.87, *p* = 0.01, eta^2^ = 0.06] with higher scores in measurement 1 (0.93 ± 0.05) than 2 (0.26 ± 0.04), and, of course, a main effect of hypnotizability [*F*(1, 100) = 7.20, *p* = 0.009, eta^2^ = 0.07] with higher values in high (0.42 +/− 0.06) than low hypnotizable participants (0.22 +/− 0.04). The interaction with hypnotizability was however non-significant [*F*(1,100) = 0.02, *p* = 0.88, eta^2^ < 0.001]. Including amnesia as a covariate did not change the results or favor another conclusion (see Supplementary material).

## 4 Discussion

In our two reported studies, the amount of experience in hypnosis did not enhance scores of hypnotizability. On the contrary, participants initially scoring high in hypnotizability revealed significantly lower scores at retest. Low hypnotizable participants did not significantly alter their scores when retested. The modality of hypnotizability (online vs. offline) did not alter overall hypnotizability scores.

Our results are in contradiction to evidence claiming that training or extended experience to hypnosis can increase hypnotizability (Kaczmarska et al., 2020). However, Bates and Brigham (1990) only found increases in the overt reactions of the subjects, observed externally, but not in the subjective scales of hypnotizability after the Carleton Skills Training Package on hypnotizability. Thus, it is possible that some training effects are masked in our study because we used only subjective reports in the HGSHS and no external ratings. It might therefore be possible that training effects on hypnotizability might be observed when using other dependent variables than the HGSHS. While subjective evaluations might be a disadvantage, the HGSHS is a highly established assessment tool for measuring hypnotizability and one of the most widely used ones also when trying to modify hypnotizability (see Acunzo and Terhune, 2021). In addition, the key elements of “alterations in physiology, sensations, emotions, thoughts, or behavior during hypnosis” (Elkins et al., 2015, p. 6) should actually be accessible by the subjective evaluations of the participants. In future studies, one could however consider to analyze sub-factors of the HGSHS, as for instance identified by Woody et al. (2005), instead of the general HGSHS score. As they found that specific subscales predicted different outcomes of hypnotizability than the general score, they argued that specific skills might add to influence overall hypnotic responses. Also, others have demonstrated that standardized scales are rather multidimensional, reducing the meaningfulness of the total score to predict responses of subjects in experimental sessions (Zahedi and Sommer, 2022). This also goes back to flaws such as guessing or compliant responding that had been identified for some of the specific suggestions in the HGSHS (Acunzo and Terhune, 2021).

Assuming that increased experience with hypnosis intensifies suggestibility, one could argue that the induction of amnesia was increased by experience, and therefore the subjective judgments of the participants were flawed during the second testing of the HGSHS. However, posthypnotic amnesia was induced only in very few participants during the second testing session, resulting in a significantly lower score in the second compared with the first testing session. In fact, posthypnotic amnesia is even more difficult to induce when participants do the HGSHS for the second time, as their repeated experience with the tasks increases the likelihood of successfully remembering the different task items. One could argue that the failure to induce posthypnotic amnesia in high hypnotizable participants might be a reason of the decrease in average hypnotizability score at retesting. However, our explorative analyses showed that there was no interaction between time and hypnotizability level, but only a main decrease over time. Moreover, including amnesia as a covariate did not change this conclusion. This supports the idea that encountering the item the second time makes it more difficult to fulfill, but excludes that this overall decline can explain our results of a specific decrease in hypnotizability scores in initially high hypnotizable participants.

One could assume that experience with hypnosis only succeeds in increasing hypnotizability if the treatment was experienced as being effective. We could operationalize this success by analyzing the subjective sleep quality reports our hypnotic intervention had targeted. Including the change in subjective sleep quality from before to after training as a covariate and correlating it with the change in HGSHS scores did not, however, confirm this assumption. This suggests that experience with hypnosis does not alter hypnotizability. Consistent with this conclusion is that neither pre-existing experience with either hypnosis or other relaxation techniques were determinants for hypnotizability. These findings do not exclude that explicit training of hypnotizability as reported for instance by Bertrand et al. (1993) or Bates and Brigham (1990) with the Carleton Skills Training Package cannot work. They had reported positive effects for hypnotizability scores after using this training package. Here, we introduced subjects to a hypnosis aiming to deepen sleep as a possibility to enlarge their degree of experience with hypnosis, but did not explicitly train hypnotizability as this package intends to do. In this context, it should however be mentioned again that the re-“exposure” during the retest showed lower HGSHS scores in initially high hypnotizable subjects. A possible explanation of the latter finding of reduced scores in high hypnotizable participants is that the repeated confrontation of subjects with hypnotic suggestions induces inner-subjective factors such as boredom, disinterest, disengagement, or reduced concentration (Barber and Calverley, 1966; Fassler et al., 2008). These factors are even more likely to influence data when the interval between the measures is rather short, such as in our experiment. When using a longer interval length (for instance, an average of 5 months as in, e.g., Palfi et al., 2020), reductions in hypnotizability were not found. We neither included longer interval lengths nor assessed subjective reports about such factors. Depending on the theoretical framework, such variables are sometimes even considered a part of the definition of hypnosis (Lynn et al., 2015). For instance, Lynn et al. (2019) recommended not defining hypnosis as a unique state, but a “broad array of alterations in consciousness” (p. 498). They argue that as a diversity of socio-cognitive factors (expectations, motivation, attitudes, beliefs) acts on the production of hypnotic responding, variability in what subjects experience during hypnosis is too large for what can be called a state (Lynn et al., 2019). Similarly, the same researchers reported that differences in responses to hypnosis can be achieved by socio-cognitive factors (Lynn et al., 2019, 2023). Whether such factors act on hypnotizability, hypnosis, or are regarded as additional factors is still a matter of debate. Another suspicious factor could be that as subjects realize the overlap between the two hypnotizability assessments, they develop the tendency to behave consistently. This would result in high associations between both behaviors and hinder changes in hypnotizability. However, to exclude or reduce this effect, Spanos et al. (1989) discussed that both measures could take place in different contexts. Even though he usually refers to supposedly two experiments which are, in reality, part of the same experiment, we had a change in context referring to the environment in which the hypnosis sessions took place. In experiment 1, we presented the hypnotic training file at home while hypnotizability was measured with a different tape at the university. In experiment 2, we measured once at home and the other assessment was at the university. In addition, these inner-factors should be present in both high and low hypnotizable participants, so they cannot fully explain why the reduction in HGSHS scores was most prominent in high hypnotizable participants. Another possible explanation is that low hypnotizable participants were not able to decrease further as they had already reached the bottom of the HGSHS scale (floor effects). However, with a mean of 4.83 ± 0.18 and 4.73 ± 0.33 in our two samples and a median of 5.00 in low hypnotizable participants, there appears to be still some room for further decreases of the average score.

Reductions in hypnotizability with repeated testing and training have already previously been reported; in Barber and Calverley (1966), reductions were found across eight individual, repeated hypnosis sessions. The authors reported reducing concentration and interest with subsequent retests. However, also in Fassler et al. (2008), with only two sessions, experience with hypnosis in the first session shaped expectancies for the second measure in a way that subjects expected lower responsiveness to hypnosis in session 2 compared to session 1. This was again discussed to be related to increased annoyance and a resulting reduced engagement. Fassler et al. (2008) reported that these inner-personal changes were present despite an increase in subjects’ positive attitudes toward hypnosis. The latter is in line with our observation of reduced hypnotizability even when the hypnotic treatment, measured as subjective sleep quality, was experienced to be successful.

A second aim of our study was to investigate the influence of online versus offline collected data on hypnotizability. As expected from previous literature, we could not detect any influence of presentation mode on hypnotizability scores. Moreover, the online and offline scores for hypnotizability and hypnotic depth were correlated to a high degree, indicating that they are strongly associated. This suggests that to facilitate screening of hypnotizability, the HGSHS can also be performed online. In our setting, this included playing the audio recording, but meeting in a group only occurred virtually in a videocall. Our findings are line with previous reports comparing online and offline assessment of hypnotizability (Palfi et al., 2020).

While the overall scores in hypnotizability scores did not significantly differ, the test–retest reliability between the two assessment modalities was lower than expected (*r* = 0.44). Previous studies reported test–retest reliability values of *r* = 0.71 across 25 years and *r* = 0.64 after 10 years (Piccione et al., 1989). For shorter intervals, the test–retest correlations between day 1 and 2 were *r* = 0.82, reducing however across the following three to eight sessions (i.e., *r* = 0.70 to *r* = 0.29) (Barber and Calverley, 1966). The correlations between the HGSHS and a short version of the questionnaire was *r* = 0.83 (Riegel et al., 2021) when the test was performed in the same modality. Also when testing the correlation between HGSHS scores in an individual vs. a group setting, the reliability coefficients were higher (*r* = 0.83) (Angelini et al., 1999). As the HGSHS is a group scale and some of the previous test intervals were quite large, some might have just over-estimated the actual reliability of the assessment. It must be considered that its reliability is simply lower than previously reported. Further studies are required to identify possible reasons for the decreased test–test-reliability between offline and online assessment of hypnotizability with the HGSHS. These future findings could be an important basis to develop a reliable online assessment of hypnotizability.

Altogether, our data shows that neither experience nor modality of presentation had an impact on hypnotizability measures. This does not exclude that explicit training of hypnotizability cannot increase responsiveness, but highlights that it is not a mere exposure effect. Our results support the notion that hypnotizability should be considered rather a stable trait (Piccione et al., 1989) than a trainable ability. Retesting highly hypnotizable subjects with the same hypnotizability measure, however, significantly reduced scores, probably uncovering the impact of additional, socio-cognitive factors. In addition, our data confirmed other reports about comparable assessments of hypnotizability in online vs. offline assessments, with some questions concerning the test–retest reliability. Both findings are relevant not only for research, where hypnotizability assessment is a critical determinant of treatment effects, but also clinically, hypnotizability could represent an important diagnostic criterion for the use of hypnotherapy. A reliable online pre-assessment could simplify the screening process. Moreover, as hypnotizability is normally distributed, the majority of people are in the middle range of hypnotizability (Elkins, 2014). In order to increase efficiency and accessibility to the benefits of hypnosis, it is of great importance to know which factors influence hypnotizability.

## Data availability statement

The raw data supporting the conclusions of this article will be made available by the authors, without undue reservation.

## Ethics statement

The studies involving humans were approved by ethical review board of the Department of Psychology, University of Fribourg. The studies were conducted in accordance with the local legislation and institutional requirements. The participants provided their written informed consent to participate in this study.

## Author contributions

BR: Conceptualization, Funding acquisition, Methodology, Writing – review & editing. MC: Conceptualization, Data curation, Formal analysis, Investigation, Project administration, Supervision, Visualization, Writing – original draft, Writing – review & editing, Funding acquisition.
