# Supplementary material for: The influence of experience and modality of presentation (online vs. offline) on hypnotizability
Source: Front Psychol. 2024 Feb 28;14:1293070. doi: 10.3389/fpsyg.2023.1293070 (PMC10936846; doi:10.3389/fpsyg.2023.1293070)
Supplement: Supplementary file 1 [file Data_Sheet_1.docx]

**Supplementary Material.**

**Results**.

**3.1 Experiment 1: Impact of experience on hypnotizability**

3x2x2 repeated measure ANOVA on the HGSHS scores with experience (hypnosis, PMR, control) and hypnotizability (high vs. low) as between-subjects factors and measurement time (pre vs. post) as within-subjects factor.

| **Effect** | **Factors** | **F statistic** | ***p* value** | **eta^2^** |
| --- | --- | --- | --- | --- |
| Main effect | Measurement time | F(1,71) = 24.09 | < .001 | .25 |
|  | Experience | F(2, 71) = 1.10 | .34 | .03 |
|  | Hypnotizability | F(1,71) = 50.37 | < .001 | .42 |
| Interaction | Measurement time * Experience | F(2, 71) = 0.55 | .58 | .02 |
|  | Measurement time * Hypnotizability | F(1, 71) = 15.45 | < .001 | .18 |
|  | Experience * Hypnotizability | F(2, 71) = 0.32 | .73 | .01 |
|  | Measurement time * Experience* Hypnotizability | F(2,71) = .01 | .99 | <.001 |

Additionally, we analyzed an ANCOVA including training success, measured as difference between PSQI after training – PSQI before training as covariate.

| **Effect** | **Factors** | **F statistic** | ***p* value** | **eta^2^** |
| --- | --- | --- | --- | --- |
| Main effect | Measurement time | F(1,63) = 17.12 | < .001 | .21 |
|  | Experience | F(2,63) = 0.55 | .58 | .02 |
|  | Hypnotizability | F(1,63) = 44.67 | < .001 | .42 |
|  | PSQI | F(1,63) = 0.42 | .52 | .007 |
| Interaction | Measurement time * Experience | F(2,63) = 0.20 | .82 | .01 |
|  | Measurement time * Hypnotizability | F(1,63) = 11.35 | .001 | .15 |
|  | Measurement time * PSQI | F(1,63) = 0.32 | .57 | .01 |
|  | Experience * Hypnotizability | F(2,63) = 0.30 | .74 | .01 |
|  | Measurement time * Experience* Hypnotizability | F(2, 63) = .002 | .99 | <.001 |

To estimate the influence of covariates, we re-analyzed the upper ANOVA including the additional between-subjects factor experience with hypnosis or experience with relaxation.

| **Effect** | **Factors** | **F statistic** | ***p* value** | **eta^2^** |
| --- | --- | --- | --- | --- |
| Main effect | Measurement time | F(1,63) = 12.62 | .001 | .17 |
|  | Experience | F(2,63) = 1.74 | .18 | .05 |
|  | Hypnotizability | F(1,63) = 46.20 | < .001 | .42 |
|  | Experience with hypnosis | F(1,63) = 0.99 | .32 | .015 |
| Interaction | Measurement time * Experience | F(2,63) = 0.84 | .44 | .03 |
|  | Measurement time * Hypnotizability | F(1,63) = 15.34 | <.001 | .20 |
|  | Measurement time * Experience with hypnosis | F(1,63) = 0.03 | .87 | <.001 |
|  | Experience * Hypnotizability | F(2,63) = 0.09 | .91 | .003 |
|  | Measurement time * Experience* Hypnotizability | F(2, 63) =0.05 | .95 | .02 |

**3.2 Experiment 2: Online vs. Offline modality of HGSHS assessment**

Moreover, we included the between-subjects factor hypnotizability as measured in the first session (high vs. low) into a 2 x 2 repeated measures ANOVA with the between-subjects factor measurement time (session 1 vs. 2). Main effect of measurement time *F*(1, 100) = 23.67, *p* < .001, eta^2^ = .19, main effect of hypnotizability *F*(1, 100) = 83.21, *p* < .001, eta^2^ = .45. Interaction *F*(1,100) = 18.94, *p* <.001, eta^2^ = .16.

**Explorative analysis.**

To test the influence of the amnesia item on the total HGSHS Scores, we included the difference in amnesia between session 2 and 1 as a covariate into a 2x2 ANCOVA (factors measurement time and hypnotizability) on the HGSHS scores of sessions 1 and 2. A positive value in the covariate would indicate a gain in amnesia scores, a negative score indicates a loss (i.e., criterion met in session 1, but not in session 2). As in the ANOVA without the covariate, we observed a main effect of time (*F*(1,99) = 18.82, *p* < .001, eta^2^ = .16), of hypnotizability (*F*(1, 99) = 82.42, *p* < .001, eta^2^ = .45) and a significant interaction between measurement time and hypnotizability (*F*(1, 99) = 19.44, *p* < .001, eta^2^ = .16). Consequently, the main effect of amnesia (*p* = .87) and its interaction with measurement time were non-significant (*p* = .11).
